# Intersections of mental health and sexual and reproductive health during the COVID-19 pandemic: A mixed-methods study of women and gender-diverse people with disabilities

**DOI:** 10.1177/17455057261466290

**Published:** 2026-08-10

**Authors:** Sidrah K. Zafar, Carmela Melina Albanese, Meredith Evans, Alexandra Rego, Keat Welsh, Nkem Ogbonna, Lucy C. Barker, Anne Berndl, Janice Du Mont, Yona Lunsky, Amy C. McPherson, Sara Rotenberg, Lesley A. Tarasoff, Ashley Vandermorris, Hilary K. Brown

**Affiliations:** 1 Department of Health and Society, 33530 University of Toronto Scarborough, Toronto, ON, Canada; 2 Dalla Lana Schol of Public Health, University of Toronto, Toronto, ON, Canada; 3 Women’s College Hospital Research Institute, Toronto, ON, Canada; 4 Reproductive Life Stages Program, 7985 Women’s College Hospital, Toronto, ON, Canada; 5 Department of Psychiatry, Temerty Faculty of Medicine, University of Toronto, Toronto, ON, Canada; 6 Sunnybrook Health Sciences Centre, Toronto, ON, Canada; 7 Department of Obstetrics and Gynecology, Temerty Faculty of Medicine, University of Toronto, Toronto, ON, Canada; 8 Sunnybrook Research Institute, Toronto, ON, Canada; 9 Azrieli Adult Neurodevelopmental Centre, Centre for Addiction and Mental Health, Toronto, ON, Canada; 10 Research Institute, Holland Bloorview Kids Rehabilitation Hospital, Toronto, ON, Canada; 11 Rehabilitation Sciences Institute, University of Toronto, Toronto, ON, Canada; 12 International Centre for Evidence in Disability, London School of Hygiene and Tropical Medicine, London, UK; 13 Division of Adolescent Medicine, The Hospital for Sick Children (SickKids), Toronto, ON, Canada; 14 Department of Paediatrics, Temerty Faculty of Medicine, University of Toronto, Toronto, ON, Canada

**Keywords:** COVID-19, mental health, persons with disabilities, qualitative research, surveys and questionnaires

## Abstract

**Background:**

The COVID-19 pandemic has had significant mental health impacts globally. However, prior studies have not examined the specific mental health effects of the COVID-19 pandemic on women and gender-diverse people with disabilities, particularly as they intersect with sexual and reproductive health (SRH).

**Objectives:**

Our objective was to examine the impacts of the COVID-19 pandemic on mental health and access to mental health care among women and gender-diverse people with disabilities, including how mental health intersected with SRH.

**Design:**

We conducted a community-engaged concurrent mixed-methods study in Canada with women and gender-diverse people with physical, sensory, psychiatric, and cognitive disabilities recruited using convenience and purposeful sampling.

**Methods:**

Between May, 2022 and March, 2023, participants completed a survey (including identification of anxiety and depression symptoms using the Patient Health Questionnaire-4) and/or in-depth semi-structured interviews. Quantitative data were analyzed using descriptive statistics, and qualitative data were analyzed using deductive and inductive coding following a constructivist approach.

**Results:**

The study included 116 survey participants and 61 interview participants, with 50 participants completing both the survey and interview. Twenty-nine per cent of participants rated their mental health as poor during the pandemic (vs. 21.6% before), and 58.3% had symptoms of anxiety and/or depression. Participants described social isolation, threat of infection, and ableism as contributing to poor mental health. Over half (54.2%) who sought mental health care reported barriers. Long wait times, inaccessible virtual care, restrictions on support persons at appointments, and economic insecurity impacted access. Mental health and SRH intersected, with pandemic-related psychological distress negatively affecting menstrual health, sexual wellbeing, perinatal health, and menopause. Delays accessing SRH care also contributed to poor mental health.

**Conclusion:**

These data show the need for integration of accessible mental health and SRH services and promotion of disability-affirming mental health care during public health emergencies and beyond.

## Plain language Summary

The COVID‑19 pandemic negatively impacted people’s mental health around the world. However, very little research has looked specifically at how the pandemic affected the mental health of women and gender‑diverse people with disabilities, especially in connection with their sexual and reproductive health (SRH). Our study explored these issues by examining how the pandemic influenced mental health, access to mental health care, and the ways mental health and SRH affected each other. Working with community partners across Canada, we recruited women and gender‑diverse people with physical, sensory, psychiatric, and cognitive disabilities to complete a survey and take part in an interview between May 2022 and March 2023. In total, 116 people completed the survey and 61 completed interviews; 50 participated in both. During the pandemic, 29.3% of participants said their mental health was poor, compared with 21.6% before the pandemic. More than half (58.3%) reported symptoms of anxiety and/or depression. Participants described social isolation, fear of getting COVID‑19, and negative attitudes from health care providers as major contributors to their distress. Among those who tried to get mental health care, over half (54.2%) faced barriers. These included long wait times, inaccessible virtual care, rules that prevented support people from attending appointments, and financial hardship. Participants also explained that mental health and SRH were closely connected. Stress and anxiety during the pandemic affected menstrual cycles, sexual wellbeing, pregnancy‑related health, and menopause. At the same time, delays in accessing SRH care worsened their mental health. Overall, our findings highlight the need for accessible mental health and SRH services, and for approaches to care that support women and gender‑diverse people with disabilities during and beyond public health emergencies.

## Introduction

The COVID-19 pandemic has had significant mental health impacts globally, including elevated rates of anxiety and depression.^1,2^ Economic and social disruptions; extensive media coverage and misinformation; and fear of infection amplified psychological distress, as well as general feelings of grief and loneliness.^3,4^ The stressors brought on by the COVID-19 pandemic coincided with disrupted psychiatric care and unmet needs for mental health supports at a time when demand for these services was heightened.^5–7^ Many countries attempted to adjust their mental health care strategies to address access barriers, such as delivering mental health services via virtual modalities.^7^ However, despite these efforts, historically underserved communities experienced disproportionate negative impacts of the pandemic on their mental health.^5^

Over 1.3 billion people globally have a disability.^8^ During the COVID-19 pandemic, people with disabilities were more likely than those without disabilities to report experiencing loneliness, anxiety, depression, suicidal ideation, and new or increasing substance use.^9–13^ The amplified mental health impacts of the pandemic among people with disabilities are thought to be partly a result of facing pandemic-related economic and psychosocial stressors at higher rates than people without disabilities.^14–22^ For example, disabled people reported greater disruptions to employment, personal and family finances, food sufficiency, and access to medical care.^14–18^ They also faced elevated rates of social isolation, reduced access to essential disability-related services, barriers to COVID-19 information and vaccinations, and heightened ableism in health care settings (e.g., critical care triage protocols that discriminated against disabled lives).^19–22^

However, studies have not examined the specific mental health impacts of the COVID-19 pandemic on women and gender-diverse people with disabilities, particularly as they intersect with sexual and reproductive health (SRH). Mental health and SRH can be intertwined. For example, gynaecological conditions, sexually transmitted infections, gender-based violence, infertility, and pregnancy complications can cause psychological distress,^23–25^ and depression, anxiety, and post-traumatic stress disorder are known to influence SRH outcomes such as sexual risk behaviours and contraceptive use.^26,27^Pre-pandemic data show women with disabilities are more likely than those without disabilities to experience disparities in both mental health^28^ and SRH outcomes and care^29–31^ and are also more likely to experience SRH-related mental health conditions such as postpartum depression and anxiety.^28^ During the COVID-19 pandemic, women with disabilities experienced unique barriers accessing SRH services, including the inability to bring support persons or sign language interpreters to SRH care appointments due to infection control procedures.^26–32^ Further investigation of the intersection between mental health and SRH for women and gender-diverse people with disabilities is warranted, as mental health data considering the gendered experiences of disability during the COVID-19 pandemic could inform psychosocial supports in future public health emergencies and beyond.

This mixed-methods study examined the impact of the COVID-19 pandemic on the mental health and mental health care experiences of women and gender-diverse people with disabilities, including how mental health intersected with SRH experiences during the pandemic.

## Methods

### Study context

In Canada, the COVID-19 pandemic saw multiple waves of infection, with the most significant incidence of hospitalizations and deaths, and corresponding lockdowns and physical distancing measures, occurring in Spring 2020, Winter 2021, and Winter 2022.^33^ Like other regions, Canada experienced increased unemployment, education disruptions, and health care strain during this time.^33^ Mass vaccination efforts began in December 2020, and most mask mandates, other than in health care facilities, largely ended by Summer 2023.^33^

### Study overview

We conducted a community-engaged concurrent mixed-methods study in Canada between May 2022 and March 2023, comprised of a survey and semi-structured interviews.^34^ We worked with disability communities by partnering with the DisAbled Women’s Network of Canada and Ase Community Foundation for Black Canadians with Disabilities, who formed an advisory committee that consulted on the study design, data collection, and interpretation. Peer researchers with disabilities were also included on the research team. Approval was obtained from the University of Toronto Research Ethics Board (protocol #42194). Our study follows the Framework for Descriptive Epidemiology guidelines^35^ in the reporting of our survey data and the Consolidated Criteria for Reporting Qualitative Research^36^ in the reporting of qualitative data.

### Data collection

Convenience sampling was used to recruit participants for the survey, with purposeful sampling used for the interviews to ensure sample diversity with respect to disability types, gender, race/ethnicity, and geographic region. Recruitment flyers and a video with closed captioning were disseminated via social media, the listservs of disability organizations, and disability advocates. Part way through recruitment, we stopped social media recruitment due to numerous fraudulent participation requests. This was a common issue for studies offering honoraria for research participation during the COVID-19 pandemic.^37^ To complete recruitment, we subsequently used only disability organizations’ email distribution lists and word-of-mouth.

Interested individuals contacted the study coordinator, who screened them for eligibility. Eligible participants were 18 years and older; were women or gender-diverse; had a self-identified physical (e.g., cerebral palsy, fibromyalgia, multiple sclerosis), sensory (e.g., vision loss, hearing loss), psychiatric (e.g., bipolar disorder, post-traumatic stress disorder), or cognitive disability (e.g., ADHD, autism, dyslexia, intellectual disability); lived and received health care in Canada; and were able to provide informed consent to participate in research. Before data collection began, a member of the research team obtained informed consent in accordance with University of Toronto Research Ethics Board guidelines, using written or verbal approaches based on participant preference. Verbal consent was offered as an accessibility measure to accommodate participants’ needs and preferences. For participants who provided verbal consent, this was documented by the research team. Participants were provided a copy of the information letter and consent form for their records. For participants with intellectual disabilities, additional questions were asked to ensure participants’ understanding of the study purpose, voluntariness, the right to skip or decline questions, and measures to protect their privacy and confidentiality.^38^

Participants could complete the survey, the interview, or both. Surveys were designed to be 20 minutes and were completed via REDCap, a secure web-based application,^39^ with the Completely Automated Public Turing test to tell Computers and Humans Apart (CAPTCHA) feature to prevent completion by bots.^40^ Participants could complete the survey online, or with the assistance of the study coordinator via Zoom/telephone. The REDCap version of the survey had a dictation feature that participants could turn on to read the questions and answer options aloud, and participants could also adjust the font size. Interviews were typically 60 minutes and were conducted by a medical anthropologist and trained peer researchers. The interviewers debriefed and recorded field notes to capture key points after the interviews.

Accommodations (e.g., sign language interpreters, ability to review a summary of the interview questions ahead of time) were provided to participants upon request, and third parties (e.g., interpreters, support persons) signed confidentiality agreements.

A safety protocol was available in case of distress (e.g., instructions on pausing interviews, determining participant wellbeing, and contacting emergency services if needed), but there were no safety concerns or withdrawals.

Participants received a $15 CAD gift card for participating in the survey and a $40 CAD gift card for participating in the interview, along with a list of resources related to disability, SRH, and mental health services.

### Measures

The survey and interview guide were developed in consultation with the study advisory committee, who reviewed the instruments for appropriateness, clarity, and accessibility. Survey items were drawn from validated instruments or adapted from existing surveys, and additional items were developed for this study. The tools were also trialed by members of the research team, including peer researchers with disabilities, prior to full implementation.

All participants completed a socio-demographics questionnaire, including questions about their disability, age, gender, sexual identity, relationship status, race, education level, employment status, and geographic region.

The survey (Supplement A) included questions about the socioeconomic impacts of the pandemic, including loss of disability-related supports, employment, income, and housing, and food insecurity.^41^ Mental health measures included self-reported mental health before and during the pandemic (rated as *poor*, *fair*, *good*, *very good*, or *excellent*) and the Patient Health Questionnaire-4 (PHQ-4),^42^ which rates the frequency of symptoms of anxiety (2 items) and depression (2 items) in the last 2 weeks on a 4-point Likert scale (*not at all*, *several days*, *more than half the days*, *nearly every day*). Each PHQ-4 subscale has a range of scores from 0 to 6, with 3 or more considered a positive screen for anxiety or depression. The PHQ-4 has been used to screen for anxiety and depression among people with disabilities,^43,44^ showing good reliability (McDonald’s ω 0.775–0.852) and excellent fit for a two-factor solution (reflecting the anxiety and depression subscales) in rehabilitation samples with physical and cognitive disabilities, for example.^44^ The survey also assessed access to mental health care, with participants reporting if they had need for and use of mental health services during the COVID-19 pandemic, and whether they experienced any barriers, delays, or disruptions seeking mental health care.

The interview guide (Supplement B) included questions about disability and SRH services, including experiences accessing, trying to access, or thinking about accessing SRH services during the COVID-19 pandemic, and recommendations for improving SRH services during and beyond public health emergencies. Follow-up questions probed the mental health impacts of the pandemic, including the specific impacts of SRH experiences on wellbeing.

### Statistical and qualitative analysis

Descriptive statistics were generated to separately describe the survey and interview participants.

Using survey data, we used descriptive statistics to report the social and economic impacts of the COVID-19 pandemic, the proportions of participants who rated their mental health as *poor* before and during the COVID-19 pandemic and who screened positive for anxiety and depression, the mental health services they accessed, and barriers, delays, or disruptions seeking mental health care. Quantitative data analysis was completed using SAS v. 9.4 (SAS Institute Inc., Cary, NC).

Interview audio-recordings were transcribed verbatim by a professional transcription agency, and participants were assigned pseudonyms to ensure anonymity. Interview data were analyzed following a constructivist approach.^45^ We applied a hybrid method to coding transcripts in NVivo 12, using deductive coding for preidentified mental health-related themes (e.g., experiences of anxiety, depression, distress) and inductive coding to map new themes related to mental health care experiences during the COVID-19 pandemic and the relationship between mental health and SRH. We triangulated the analysis by having multiple researchers analyze the data and by consulting with the broader research team and the advisory committee to refine our interpretation.^46^ Member checking was not undertaken due to methodological orientation (the qualitative analysis was treated as an interpretive process in which findings are understood as situated constructions rather than accounts requiring participant validation), and to minimize further demands on participants’ time.^47^

### Sample size

We did not complete an *a priori* sample size calculation for the survey, given that we used convenience sampling and could not make statistical inferences to the larger population.^48^ Instead, we aimed for as large a sample as possible, with attention to diversity across variables of relevance, such as disability type.

For the interviews, data adequacy was assessed via ongoing engagement with the dataset, considering whether the interview data offered sufficient depth and nuance to address the research question.^49^ We evaluated whether the developing themes were well-supported by the data and analytically rich across factors such as disability type. Data collection was complete when additional interviews no longer contributed meaningfully to the analysis.^49^

## Results

### Sample characteristics

The study included 116 survey participants and 61 interview participants, with 50 individuals completing both the survey and interview (Table 1). Regarding disability type, participants self-reported physical (64.7%), cognitive (57.8%), psychiatric (33.6%), and sensory disabilities (14.7%), with 46.6% having multiple disabilities. Participants had a median age of 32 years (range 18-63). Most identified as heterosexual (57.8%), cisgender women (79.3%), and were in a relationship (55.6%). Most were white (67.5%), and half had a post-secondary degree (50.9%) and were working full-time or part-time for pay (46.5%). Almost half lived in a city with a population of 500,000 or more (44.4%), and most lived in Ontario (69.8%). The characteristics of the interview participants largely mirrored those of the survey participants, except that a slightly greater proportion of interview participants had psychiatric disabilities and were single and Black, and a smaller proportion were students (Table 1).

**Table 1.** Participants’ sociodemographic characteristics. Data presented as n (%) unless otherwise specified.

Characteristic	Categories	Survey participants (N=116)	Interview participants (N=61)
Disability*	Physical	75 (64.7)	41 (67.2)
	Sensory	17 (14.7)	6 (9.8)
	Psychiatric	39 (33.6)	27 (44.3)
	Cognitive	67 (57.8)	26 (42.6)
Age (years)^†^	18-24	24 (20.9)	18 (29.5)
	25-34	43 (37.4)	20 (32.8)
	35-49	34 (29.6)	17 (27.9)
	50-64	14 (12.2)	6 (9.8)
Gender identity*	Cisgender woman	92 (79.3)	48 (78.7)
	Transgender man, transgender woman, genderqueer, non-binary, Two-Spirit, other	24 (20.7)	15 (24.6)
Sexual identity	Heterosexual/straight	67 (57.8)	36 (59.0)
	Gay/lesbian, queer, bisexual, pansexual, other (e.g., questioning)	49 (42.2)	25 (41.0)
Relationship Status^‡^	Partnered (married, common-law, long-term relationship)	64 (55.7)	29 (47.5)
	Single (never married, separated, widowed, divorced)	51 (44.3)	32 (52.5)
Racial background*^§^	Asian, East Asian, South Asian, Pacific Islander, Middle Eastern	33 (28.4)	9 (14.8)
	Black, Afro-Caribbean, Afro-Indo Caribbean, African	16 (13.8)	13 (21.3)
	Indigenous	8 (6.9)	3 (4.9)
	White	79 (67.5)	40 (65.6)
Highest level of education	High school or less	21 (18.1)	12 (19.7)
	College/trade diploma	36 (31.0)	23 (37.7)
	Undergraduate degree	42 (36.2)	18 (29.5)
	Graduate degree	17 (14.7)	8 (13.1)
Employment status at data collection*	Working full-time for pay	37 (31.9)	20 (32.9)
	Working part-time for pay	20 (17.2)	9 (14.8)
	Student	29 (25.0)	8 (13.1)
	On medical or disability leave	15 (12.9)	10 (16.4)
	Other (seeking employment, retired, caring for family, volunteering)	18 (15.5)	14 (23.0)
Geography of residence^¶^	Rural/small town (<1,000 or 1,000-29,999)	12 (10.4)	6 (9.8)
	Medium-sized city (30,000-99,999)	24 (20.9)	8 (13.1)
	Big city (100,000-499,999)	28 (24.3)	22 (36.1)
	Very big city (≥500,000)	51 (44.3)	25 (41.0)
Province/territory of residence	Atlantic Canada (Nova Scotia, New Brunswick, Prince Edward Island, Newfoundland and Labrador)	4 (3.5)	2 (3.3)
	British Columbia	13 (11.2)	6 (9.8)
	Ontario	81 (69.8)	45 (73.8)
	Prairies (Alberta, Saskatchewan, Manitoba)	14 (12.1)	7 (11.5)
	Québec	2 (1.7)	1 (1.6)
	Territories (Northwest Territories, Nunavut, Yukon)	2 (1.7)	0 (0.0)

*Categories are not mutually exclusive, so total sum of all categories exceeds 100%.

Missing data by characteristic: †N=1; ‡N=1; §N=1; ¶N=1.

One-third (29.3%) of survey participants reported a loss of help with their daily activities during the pandemic; more than half (59.3%) had a partial or total loss of income; and many (23.7%) had a partial or total loss of employment and greater food insecurity (47.4%) (Table 2).

**Table 2.** Social and economic impact of the COVID-19 pandemic on women and gender-diverse people with disabilities, from among n=116 survey participants.

Characteristic	Categories	N (%) with outcome
Changes in receipt of help with daily activities	None	58 (50.0)
	Less help than before COVID-19	34 (29.3)
	More help than before COVID-19	24 (20.7)
Loss of household income^†^	None	35 (31.0)
	Partial loss during COVID-19	53 (46.9)
	Total loss during COVID-19	14 (12.4)
	Not applicable (no personal income before pandemic)	11 (9.7)
Changes in work status^††^	None	26 (23.6)
	Partial loss of work during COVID-19	6 (5.5)
	Total loss of work during COVID-19	20 (18.2)
	Not applicable (not working before pandemic)	2 (1.8)
	Other change (e.g., keep doing the same work but from/not from home)	56 (50.9)
Food insecurity^§^	No food insecurity during COVID-19	60 (52.6)
	Food insecurity during COVID-19	54 (47.4)

Missing data by characteristic: †N=3, ††N=6, §N=2.

*Note.* Data are reported by participants during the COVID-19 pandemic up to the point of data collection. Where losses are described, these are in comparison to the three-month period prior to the pandemic.

### Mental health impacts of the COVID-19 pandemic

**Figure 1.**
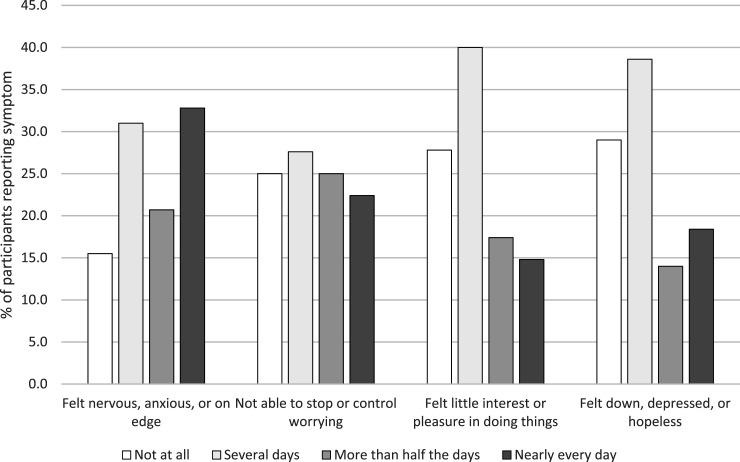
Symptoms of anxiety and depression reported by women and gender-diverse people with disabilities in the past 2 weeks during the COVID-19 pandemic, from among n=116 survey participants.

Survey data indicated that more women and gender-diverse people with disabilities felt their mental health was poor during the COVID-19 pandemic (29.3%) than before (21.6%). Symptoms of anxiety and depression were commonly reported in the last 2 weeks: 32.8% felt nervous, anxious, or on edge; 22.4% were not able to stop or control worrying; 14.8% felt little interest or pleasure in doing things; and 18.4% felt down, depressed or hopeless nearly every day (Figure 1). In total, 32.7% of survey participants screened positive for both anxiety and depression, 21.2% for anxiety only, 4.4% for depression only, and 41.7% for neither.

In the interviews, many participants described how the pandemic had an adverse impact on their mental health:

“I struggled with anxiety and depression before, and it’s worsened. I’m back on a lot of medications now, because I wasn’t able to sleep during the pandemic. I think it was just that out-of-control feeling. My partner lost his job in the first year of the pandemic [and received a chronic illness diagnosis]. I lost my mind – wasn’t eating, wasn’t sleeping.” – Tanya (Indigenous Two-Spirit person, physical and psychiatric disabilities)

“Everyone lost their minds in the pandemic. I genuinely lost my mind. My [obsessive compulsive disorder] got so bad. I once went four days without eating because I did not realize that time was passing. I just stared at a wall for three solid days.” – Sade (Black cisgender and questioning woman, cognitive, physical, and psychiatric disabilities)

“A lot of people’s mental health has been negatively impacted. It’s a different feeling overall. People are feeling more overwhelmed, more exhausted, and more financially depleted and are really struggling in a lot of different ways.” – Cameron (white genderqueer person, psychiatric disabilities)

Social isolation was a common experience for people with disabilities during the pandemic, and this worsened many participants’ mental health and wellbeing.

“I had depression for a long time, especially during the pandemic. A lot of it was the lack of connection with others. I felt the pressure of the isolation during the pandemic which did increase my depressive symptoms. I ended up having to go on medications a couple times during the pandemic.” – Madison (white cisgender woman, cognitive disabilities)

“There’s a [Canadian Mental Health Association-run] centre in my community that provides social and recreational opportunities for people with mental illnesses. They’ve closed their groups and programming. I used to go there and do creative projects weekly. […] Not having a routine has been hard.” – Emily (white cisgender woman, cognitive, physical, and psychiatric disabilities)

“The COVID-19 experience further disenfranchised me from my culture. I feel like I’m able to connect and witness and engage with my culture through being outdoors and in community.” Jody (Black, non-binary person, psychiatric disabilities)

This social isolation was exacerbated, for many participants, by the threat of COVID-19 exposure and risk of infection.

“The pandemic was awful, especially as an immunocompromised person because I really felt like I couldn’t go anywhere. […] It was very lonely, I felt completely boxed in, my job sucked, and it was really hard on my mental health.” – Rowan (white genderqueer person, physical and psychiatric disabilities)

“I don’t want [COVID or long COVID]. I have enough chronic illnesses. I don’t want another one.” – Jamie (white cisgender woman, cognitive and physical disabilities)

Others commented about how experiences of ableism during the pandemic adversely impacted their mental health.

“It’s horrible to feel like other people don’t care about your safety. […] You deal with that as a disabled person, but the magnitude at which this developed [during the pandemic] was really overwhelming, and it impacted my mental health. – Cynthia (white cisgender woman, physical and psychiatric disabilities)

On the other hand, a few participants commented that social isolation and government benefits like the Canada Emergency Response Benefit had unexpected mental health benefits.

“I started working from home. And the government’s giving me money to stay home. It was weird. It’s okay that I go for a walk in the middle of the afternoon. I can go garden in the middle of the afternoon. That’s what I did. […] It was great. It helped my mental health.” – Jamie (white cisgender woman, cognitive and physical disabilities)

### Access to mental health care during the COVID-19 pandemic

Most survey participants (82.8%) reported wanting or needing mental health services during the COVID-19 pandemic, and many participants (77.6%) used mental health services during the pandemic, including talk therapy or counseling (58.6%), psychiatrists (38.8%), family doctors or nurses (36.2%), medications (34.5%), community/student/walk-in clinics (10.3%), hospital or residential mental health treatment programs (2.6%), and other services (8.6%). Many of these services were accessed virtually (e.g., counselling) (Table 3).

**Table 3.** Use of mental health care during the COVID-19 pandemic, from among n=116 survey participants.

Type of mental health service	Total used service N (%)	Used in-person only N (%)	Used virtually only N (%)	Used in-person and virtually N (%)
Any mental health service^†^	90 (77.6)	4 (3.4)	41 (35.3)	44 (37.9)
Mental health specialist (e.g., psychiatrist)	45 (38.8)	5 (4.3)	26 (22.4)	14 (12.1)
Family doctor or nurse^†^	42 (36.2)	4 (3.4)	19 (16.4)	18 (15.5)
Community, student, or walk-in clinic	12 (10.3)	2 (1.7)	8 (6.9)	2 (1.7)
Medication	40 (34.5)	17 (14.7)	10 (8.6)	13 (11.2)
Talk therapy or counselling services^††^	68 (58.6)	2 (1.7)	47 (40.5)	17 (14.7)
Hospital or residential mental health treatment program	3 (2.6)	3 (2.6)	0 (0.0)	0 (0.0)
Other services (e.g., emergency department, distress centre, support worker, other medical specialist, telephone help line)	10 (8.6)	2 (1.7)	7 (6.0)	1 (0.9)

Missing data for mode of use by service: †N=1, ††N=2.

Over half (54.2%) of survey participants who reported wanting or needing mental health care reported barriers to services, including long lines or waiting times at clinics or health centres (55.8%), unavailable appointments with doctors or health centres (61.5%), not being able to afford mental health care or medication (40.4%), fear of contracting COVID-19 infection (36.5%), inaccessible online or telephone appointments (34.6%), absence of regular supports or accommodations needed to access services (28.8%), clinic and health centre closures (28.8%), inability to leave the house (26.9%), and unavailable transportation (19.2%). Of those who wanted or needed mental health care, 33.3% were delayed in accessing services, and 12.5% were completely prevented from obtaining care.

Interview participants also reported that long wait times during the pandemic were a deterrent to accessing mental health services.

“I wanted to get reconnected [to counselling but] everyone was trying to book in because of rising mental health issues. […] For awhile I just gave up and stopped seeking counselling.” – Logan (East/Southeast Asian transgender man, cognitive disabilities)

“I’ve been waiting for an adult mental health referral [for over four months]. They said six weeks. […] I’m panicking now because no one’s phoned me, and I don’t know who to phone.” – Tanya (Indigenous Two-spirit woman, physical and psychiatric disabilities)

Telehealth was a useful way of accessing mental health care for some participants; however, many described virtual modalities as inaccessible or not ideal.

“I had a lot of telehealth appointments for my mental health. I love telehealth, but […] it has its time and place. […] People with disabilities have been wanting [telehealth] for so long. […] It’s been wonderful to have the option because sometimes it’s hard to get out of the home. Virtual appointments make it super accessible.” – Cynthia (white cisgender woman, physical and psychiatric disabilities)

“My usual therapist wasn’t taking in-person meetings. But my room is close enough [to others]. I don’t feel comfortable talking about this while my parents are here [in the home]. So, I slowly lost my sanity because I can’t access anything. There were online resources, but you just need to talk to a person in real life.” – Sade (Black cisgender and questioning woman, cognitive, physical, and psychiatric disabilities)

Restrictions on support persons and ableist provider attitudes contributed to accessibility barriers to mental health services during the COVID-19 pandemic.

“I have a lot of medical trauma. Not being allowed to bring anyone with me, to an appointment or if I’m in the emergency room or when I’ve had to stay overnight, has made it really difficult—because that helps me. There have been a few times I’ve been in the ICU [for mental health and suicide attempts] and no one has been allowed to visit me. I had a few anxiety attacks, and it made my mental health worse. […] During COVID, [hospital staff] spent less time with you. Before the pandemic, staff would come and sit and talk. During COVID, it’s like they just come and do what they have to do, and that’s it. Then, not being able to get out of your room to go for a walk, even just in the hallway, or not being able to go down to the lobby or cafeteria. It makes such a big difference for your mental health, just being able to get out of that tiny little room.” – Isabelle (white cisgender woman, physical and psychiatric disabilities)

“The nurse is like, ‘Why don’t you go back home and get your iPad [for video ASL interpreting services]?’ when I’m having suicidal thoughts. I’m like, ‘Are you serious?’ I was just baffled. What if I had left, and harmed myself, killed myself? […] I’m scared to go into the hospital for my mental health issues, because of these communication barriers. […] [The COVID-19 pandemic] makes me very anxious, it gives me anxiety. It’s not safe for me as a Deaf person.” – Shannon (white cisgender Deaf woman)

Socioeconomic barriers aggravated by the COVID-19 pandemic also made it difficult to access mental health medications and services.

“I was rationing [my anti-depressants]. I had to stop going [to the psychiatrist] because I’m like, ‘I don’t have the money to keep going here.’ It was a two-hour bus ride, so every time I went, I had to pay an extra fare, $4 extra. It was crazy. I had to pay there and back, so that’s $8 I’m spending on top of my regular fare. I’m like, ‘I don’t have the money for this all the time.’ I just stopped going.” – Kassie (Black cisgender woman, cognitive and psychiatric disabilities)

### Intersections between mental health and SRH

Some interview participants perceived pandemic-related psychological distress to have a negative impact on SRH outcomes, such as menstrual health and sexual wellbeing.

“With the stress of the pandemic, the body reflects that mental stress. For the course of the pandemic, I’ve been noticing that when I do have my period it’s a lot heavier, and my symptoms are more pronounced.” – Ava (white genderqueer person, cognitive, physical, and psychiatric disabilities)

“[The increase in depression and anxiety I experienced during the pandemic] dropped my libido significantly. […] The weight gain associated with the pandemic too—the depression and then the weight gain affected my reproductive system, impacted my menstrual cycles.” – Madison (white cisgender woman, cognitive disabilities)

Pandemic-related stressors also exacerbated perinatal mental health concerns, like postpartum depression.

“I had to go back to work after six months [while experiencing postpartum depression]. I couldn’t afford to be home any longer because [during the pandemic] service industry jobs like [my partner’s] were shut down for pretty much two years. That wasn’t a part of the plan. [Going back to work as the sole household earner] put a lot of strain on me [postpartum]. It was really challenging emotionally and mentally. I just did not have the capacity” – Bailey (Black cisgender woman, physical and psychiatric disabilities)

During the pandemic, SRH-related experiences (e.g., pregnancy, menopause, gender-based violence) also impacted participants’ mental health.

“During COVID, I had an abortion. It was really hard going through the process. Afterwards, I dealt with severe mental health issues. I experienced severe depression.” – Farah (South Asian cisgender woman, cognitive and psychiatric disabilities)

“[The hysterectomy and menopause I experienced during the pandemic] triggered [depression and anxiety], as well as exasperated the longer-term depressive disorder I have [and my] posttraumatic stress disorder. […] COVID meant there was no support. [During COVID] I made strides not to kill myself, which after going through menopause became a reality—wanting to kill myself. And I’m still here.” – Alanna (Black cisgender woman, physical and psychiatric disabilities)

“Before I was divorced, I was struggling with mental health. [My therapist] counselled me to get out of the relationship [with my abusive ex-husband]. The beginning of the pandemic, [the abuse] got really bad. Now [that I left my ex-husband] I’m not as scared. Before, I was so worried and scared.” – Lena (white cisgender Deaf woman)

Delays accessing SRH services during the pandemic (e.g., gynaecological, pregnancy, menopause, and gender-affirming care) amplified mental health concerns such as anxiety and suicidality.

“[Delays to gynaecological care] really impacted my mental health because I was constantly worried that I’m sitting here with a cervix full of cancer and nobody is doing anything about it. […] It makes me very paranoid. […] It affects my depression. It affects my Crohn’s because it causes me stress […] it sets my anxiety off.” – Melissa (white cisgender woman, cognitive, physical and psychiatric disabilities)

“When the ultrasound place cancelled my [prenatal] appointment, my anxiety spiked, went through the roof. […] Having to wait for these appointments, having these barriers has increased my anxiety.” – Nicole (white cisgender woman, physical and psychiatric disabilities)

“Waiting for top surgery [due to pandemic-related delays] almost took my life.” – Avery (Black nonbinary transgender man, physical and psychiatric disabilities)

Changes to SRH service delivery during the pandemic, such as restrictions on support persons, also contributed to poor perinatal mental health.

“[The possibility that I couldn’t have my spouse accompany me to hospital for the birth] was all I could focus on. […] Leading up to this every appointment, I would ask the nurses, ‘what’s the policy on partners?’ I needed to mentally know and prepare, because I don’t have hands and I’m by myself and I know that you don't expect the health care system to be by your side 24/7 when you have a new baby. It was a lot of fear. It was hard.” – Megan (white, cisgender woman, physical disabilities)

“My partner never got to come to any appointments with me. Not even one. Not ultrasound, nothing. And then even when I gave birth, my partner wasn’t allowed to come into the OR with me. [Pregnancy and birth during the pandemic] was really tough. [It had] a negative impact on me and my partner […] we’re not together anymore [in part because of the] strain and stress that we had to try to navigate through COVID.” – Bailey (Black cisgender woman, physical and psychiatric disabilities)

## Discussion

### Summary of findings

This mixed-methods study of women and gender-diverse people with disabilities found that mental health was negatively impacted during the COVID-19 pandemic. Participants reported new and worsening psychological distress during the pandemic, and described social isolation, threat of COVID-19 exposure, and ableism as contributing factors. Over half of participants who sought mental health care during the pandemic reported barriers, many of which delayed or completely prevented access to services. Long wait times, inaccessible virtual care, restrictions on support persons at appointments, and economic problems impacted access. Mental health and SRH intersected in many ways, with pandemic-related psychological distress having a perceived negative impact on menstrual health and sexual wellbeing and worsening perinatal mental health concerns. Mental health was also impacted by SRH-related experiences such as pregnancy, menopause, and gender-based violence as well as COVID-related delays and restrictions to SRH services like gynaecological, pregnancy-related, and gender-affirming care.

### Comparison to previous research

Previous surveys in Canada and the US have shown that people with disabilities were more likely than those without disabilities to report psychological distress and suicidality during the COVID-19 pandemic, with one study showing that 57% of adults with disabilities reported symptoms of anxiety or depression versus 29% in those without disabilities.^9–15^ People with disabilities were also more likely than their non-disabled peers to experience barriers accessing mental health counselling and psychotropic medications during the pandemic.^9–15^ Prior research suggests that social isolation and discrimination were important contributors to poor mental health, and long wait times for services, fear of contracting COVID-19 infection when attending appointments, and the inability to bring support persons to appointments were barriers to mental health care for people with disabilities.^9–15^ Our study was not designed to compare people with and without disabilities. However, it offers insight into the distinct mental health experiences of women and gender-diverse people with disabilities. Many of the mental health concerns reported in prior research^9–15^ were echoed in our findings, alongside additional gendered dimensions, such as the impact of COVID-19 pandemic on postpartum mental health and wellbeing during menopause, that have received less attention in this population.

Our findings also add to the limited literature on the impacts of the COVID-19 pandemic on SRH in people with disabilities. Survey studies in the US and internationally found women with disabilities were twice as likely as those without disabilities to report disruptions to SRH services, including cervical cancer screening and family planning services.^32,50–53^ Service limitations and closures, fear of contracting COVID-19 infection in health care settings, and restrictions to support persons at appointments have been identified by our team^53^ and others^50–52^ as significant barriers to SRH care for people with disabilities during the pandemic. Our study builds on these data by showing the important ways in which SRH and mental health concerns intersected for women and gender-diverse people with disabilities during the COVID-19 pandemic, with pandemic-related psychological distress having a perceived negative impact on SRH experiences, and COVID-related barriers to SRH services affecting mental health.

### Implications

Our data on the impacts of the COVID-19 pandemic on mental health and access to mental health care among women and gender-diverse people with disabilities, including how mental health and SRH intersected, have important clinical implications. We show the need for integration of mental health and SRH services, including training for both mental health and SRH care providers to understand the interconnectedness of these health concerns; cross-training of staff from different disciplines to enhance their ability to recognize and provide appropriate referrals for mental health and SRH needs; co-location of psychiatric and SRH services; and the provision of person-centred, trauma-informed care.^54,55^ Service barriers experienced by women and gender-diverse people with disabilities during the COVID-19 pandemic also emphasize the importance of prioritizing accessible mental health services for individuals with disabilities during the acute phase of future public health emergencies and, even outside of acute phases, providing accessible care to people with disabilities that protects them from circulating infectious diseases. Virtual mental health care may be an option, but as described by some participants, there are drawbacks for some groups. The COVID-19 pandemic laid bare pre-existing mental health disparities for people with disabilities; lessons learned from the pandemic can inform approaches to enhance equity and inclusion in mental health care for women and gender-diverse people with disabilities going forward, including at the intersection of SRH outcomes and needs.

## Limitations

Several limitations should be considered. Our recruitment strategy was impacted by fraudulent participation requests. We therefore had to cease social media recruitment and rely on disability organizations’ email distribution lists and word-of-mouth to identify participants, potentially limiting our ability to accrue a larger sample. While other studies during the COVID-19 pandemic have reported similar issues,^37^ our convenience sampling approach may have introduced a selection bias, resulting in a sample that might not represent the broader population of women and gender-diverse people with disabilities in Canada. The results may reflect individuals who are already connected to disability organizations or related advocacy networks. Thus, the survey findings may not generalize to the wider disability population.

Our survey and interview samples included people with physical, sensory, psychiatric, and cognitive disabilities. A sizeable portion of the study sample therefore included people with pre-existing mental health concerns; however, both the survey and interview data suggest that mental health worsened for this group and was also significantly impacted among individuals with other types of disabilities. Some restrictions to the diversity of our samples should also be considered. For example, only a small number of d/Deaf people and people with vision impairments completed the study. Within the cognitive disability group, there were also few people with intellectual disabilities specifically. Despite our efforts to use accessible recruitment and data collection approaches, barriers to participation may have remained for these groups. Likewise, Indigenous Peoples, Black and other racialized individuals, gender-diverse people, and people living in rural and remote areas and outside of Ontario were underrepresented.

We did not include a comparison group of women and gender-diverse people without disabilities, or cisgender men with disabilities. However, our aim was to describe, in depth, the mental health experiences of women and gender-diverse people with disabilities during the COVID-19 pandemic. Future studies could examine how these experiences compare to other population groups.

Our quantitative mental health questions using the PHQ-4 only measured recent symptoms of anxiety and depression, not other symptoms of mental illness. The PHQ-4^42^ is a short screening tool with only four questions (two for anxiety and two for depression) that measures symptoms during the last two weeks. Although it is frequently used in surveys and is valid for people with disabilities,^43,44^ it provides a limited indication of symptom presence and severity, and is less sensitive to complexity, chronicity, and fluctuation over time than clinical assessment or more comprehensive instruments (e.g., PHQ-9, GAD-7). It also excludes other mental health conditions, meaning it does not capture the full range of mental health concerns experienced by individuals during the COVID-19 pandemic. This limitation is notable when compared to our qualitative findings, which identified severe psychological distress, such as worsening obsessive-compulsive disorder, medical trauma, and suicidality. Future studies should use more in-depth quantitative measures to fully describe the mental health impacts of the COVID-19 pandemic on women and gender-diverse people with disabilities.

Data collection occurred between May, 2022 and March, 2023. While this provided the advantage of allowing participants to reflect on more than two years of the COVID-19 pandemic, screening for anxiety and depression using the PHQ-4 (which asks about symptoms in the last two weeks) was done after the pandemic’s acute phase. We were unable to measure mental health impacts during the earlier pandemic periods (e.g., 2020 lockdown) and likely underestimated the peak mental health burden during this important period. Conversely, our qualitative data suggest important impacts of these early pandemic phases specifically.

## Conclusion

Survey and interview data from women and gender-diverse people with disabilities in Canada showed that mental health was significantly impacted during the COVID-19 pandemic, with pandemic-related psychological distress negatively affecting SRH outcomes, and experiences with SRH and related service delays negatively impacting mental health.

## Supplemental material

Supplemental material - Intersections of mental health and sexual and reproductive health during the COVID-19 pandemic: A mixed-methods study of women and gender-diverse people with disabilities Supplemental material for Intersections of mental health and sexual and reproductive health during the COVID-19 pandemic: A mixed-methods study of women and gender-diverse people with disabilities by Sidrah K. Zafar, Carmela Melina Albanese, Meredith Evans, Alexandra Rego, Keat Welsh, Nkem Ogbonna, Lucy C. Barker, Anne Berndl, Janice Du Mont, Yona Lunsky, Amy C. McPherson, Sara Rotenberg, Lesley A. Tarasoff, Ashley Vandermorris and Hilary K. Brown in Women’s Health.

Supplemental material - Intersections of mental health and sexual and reproductive health during the COVID-19 pandemic: A mixed-methods study of women and gender-diverse people with disabilities Supplemental material for Intersections of mental health and sexual and reproductive health during the COVID-19 pandemic: A mixed-methods study of women and gender-diverse people with disabilities by Sidrah K. Zafar, Carmela Melina Albanese, Meredith Evans, Alexandra Rego, Keat Welsh, Nkem Ogbonna, Lucy C. Barker, Anne Berndl, Janice Du Mont, Yona Lunsky, Amy C. McPherson, Sara Rotenberg, Lesley A. Tarasoff, Ashley Vandermorris and Hilary K. Brown in Women’s Health.

## Data Availability

The data are not publicly available due to ethical restrictions, as the informed consent obtained from participants did not permit public sharing of their data.*

## References

[1] World Health Organization . Mental health and COVID-19: Early evidence of the pandemic’s impact. World Health Organization, 2022. https://www.who.int/publications/i/item/WHO-2019-nCoV-Sci_Brief-Mental_health-2022.1

[2] United Nations . Policy brief: COVID-19 and the need for action on mental health. UN, 2020. Accessed 20 December 2024 from. https://unsdg.un.org/sites/default/files/2020-05/UN-Policy-Brief-COVID-19-and-mental-health.pdf

[3] Salenti G Peter N Tonia T , et al. The impact of the COVID-19 pandemic and associated control measures on the mental health of the general population: A systematic review and dose-response meta-analysis. Ann Intern Med 2022; 175(11): 1560–1571. 36252247 10.7326/M22-1507 PMC9579966

[4] Chiesa V Antony G Wismar M , et al. COVID-19 pandemic: Health impact of staying at home, social distancing and ‘lockdown’ measures—A systematic review of systematic reviews. J Public Health 2021; 43(3): e462–e481. 10.1093/pubmed/fdab102 PMC8083256 33855434

[5] Byrne A Barber R Lim CH . Impact of the COVID-19 pandemic – a mental health service perspective. Progress Neurol Psychiatr 2021; 25(2): 27–33. 10.1002/pnp.708

[6] Ahmedani BK Yeh H-H Penfold BB , et al. Psychotherapy disruptions before and after the transition to virtual mental health care induced by the COVID-19 pandemic. Psychiatr Serv 2024; 75(2): 108–114. 10.1176/appi.ps.20230181 37817579 PMC11881780

[7] Li H Glecia A Kent-Wilkinson A , et al. Transition of mental health service delivery to telepsychiatry in response to COVID-19: A literature review. Psych Quart 2021; 93(1): 181–197. 10.1007/s11126-021-09926-7 PMC8185490 34101075

[8] World Health Organization . Disability. World Health Organization, 2023.

[9] Czeisler ME Board A Thierry JM , et al. Mental health and substance use among adults with disabilities during the COVID-19 pandemic – United States, February-March 2021. MMWR 2021; 70(34): 1142–1149. 10.15585/mmwr.mm7034a3 34437518 PMC8389385

[10] Pettinicchio D Maroto M Chai L , et al. Findings from an online survey on the mental health effects of COVID-19 on Canadians with disabilities and chronic health conditions. Disabil Health J 2021; 14(3): 101085. 10.1016/j.dhjo.2021.101085 33744158 PMC9760304

[11] Okoro CA Strine TW McKnight-Eily L , et al. Indicators of poor mental health and stressor during the COVID-19 pandemic, by disability status: A cross-sectional analysis. Disabil Health J 2021; 14(4): 101110. 10.1016/j.dhjo.2021.101110 33962896 PMC8436151

[12] Rosencrans M Arango P Sabat C , et al. The impact of the COVID-19 pandemic on the health, wellbeing, and access to services of people with intellectual and developmental disabilities. Disabil Health J 2021; 114: 103985. 10.1016/j.ridd.2021.103985 PMC9758885 34049229

[13] Lake JK Jachyra P Volpe T , et al. The wellbeing and mental health care experiences of adults with intellectual and developmental disabilities during COVID-19. J Mental Health Res Intellec Disabil 2021; 14(3): 285–300. 10.1080/19315864.2021.1892890

[14] Wang K Manning RB Bogart KR , et al. Predicting depression and anxiety among adults with disabilities during the COVID-19 pandemic. Rehab Psychol 2022; 67(2): 179–188. 10.1037/rep0000434 35084914

[15] Ciciurkaite G Marquez-Velarde G Lewis Brown R . Stressors associated with the COVID-19 pandemic, disability, and mental health: Considerations from the Intermountain West. Stress Health 2021; 38(2): 304–317. 10.1002/smi.3091 34382736 PMC8420204

[16] Friedman C . Financial hardship experienced by people with disabilities during the COVID-19 pandemic. Disabil Health J 2022; 15(4): 101359. 10.1016/j.dhjo.2022.101359 35835660 PMC9220755

[17] Assi L Deal JA Samuel L , et al. Access to food and health care during the COVID-19 pandemic by disability status in the United States. Disabil Health J 2022; 15(3): 101271. 10.1016/j.dhjo.2022.101271 35151597 PMC8767932

[18] Lewis Brown R Ciciurkaite G . Precarious employment during the COVID-19 pandemic, disability-related discrimination, and mental health. Work Occupations 2022; 50(2): 167–187. 10.1177/07308884221129839

[19] Kavanagh A Hatton C Stancliffe RJ , et al. Health and healthcare for people with disabilities in the UK during the COVID-19 pandemic. Disabil Health J 2022; 15(1): 101171. 10.1016/j.dhjo.2021.101171 34330683 PMC8285926

[20] Koon LM Greiman L Schulz JA , et al. Examining the effects of the COVID-19 pandemic on community engagement for people with mobility disabilities. Disabil Health J 2022; 15(S1): 101212. 10.1016/j.dhjo.2021.101212 34531174 PMC8418869

[21] Steptoe A Di Gessa G . Mental health and social interactions of older people with physical disabilities in England during the COVID-19 pandemic: a longitudinal cohort study. Lancet Public Health 2021; 6(6): 365–373. 10.1016/S2468-2667(21)00069-4 PMC8517412 33894138

[22] Lewis BR Ciciurkaite G . Disability, discrimination, and mental health during the COVID-19 pandemic: Stress process model. Soc Mental Health 2022; 12(3): 215–229. 10.1177/21568693221115347 PMC9379597 38603117

[23] White SJ Sin J Sweeney A , et al. Global prevalence and mental health outcomes of intimate partner violence among women: A systematic review and meta-analysis. Trauma Violence Abuse 2023; 25(1): 494–511. 10.1177/15248380231155529 36825800 PMC10666489

[24] Zaks N Batuure A Lin E , et al. Association between mental health and reproductive system disorders in women: A systematic review and meta-analysis. JAMA Netw Open 2023; 6(4): e238685. 10.1001/jamanetworkopen.2023.8685 37071426 PMC10114079

[25] Figueiredo de Paula Eduardo JA Goncalvez de Rezende M Rossi Menezes P , et al. Preterm birth as a risk factor for postpartum depression: A systematic review and meta-analysis. J Affect Disord 2019; 259(1): 392–403. 10.1016/j.jad.2019.08.069 31470184

[26] Stevens M Ratheesh A Watson A , et al. Rates, types and associations of sexual risk behaviours and sexually transmitted infections in those with severe mental illness: A scoping review. Psychiatry Res 2020; 290: 112946. 10.1016/j.psychres.2020.112946 32450411

[27] McCloskey LR Wisner KL Cattan MK , et al. Contraception for women with psychiatric disorders. Am J Psychiatry 2021; 178(3): 247–255. 33167674 10.1176/appi.ajp.2020.20020154

[28] Deierlein AL Park C Patel N , et al. Mental health outcomes across the reproductive life course among women with disabilities: A systematic review. Arch Women’s Mental Health 2024; 28(4): 647–664. 10.1007/s00737-024-01506-5 PMC12283890 39222078

[29] Naghdi-Dorabati P Shahali S Montazeri A , et al. The unmet need for sexual health services among women with physical disabilities: A scoping review. J Public Health 2024; 33: 2163–2174. 10.1007/s10389-024-02191-5

[30] Besoain-Saldana A Bustamante-Bravo J Rebolledo Sanhueza J , et al. Experiences, barriers, and facilitators to sexual and reproductive health care access of people with sensory impairments: A scoping review. Sex Disabil 2023; 41: 411–449. 10.1007/s11195-023-09778-y

[31] Ransohoff JI Sujin Kumar P Flynn D , et al. Reproductive and pregnancy health care for women with intellectual and developmental disabilities: A scoping review. J Appl Res Intellect Disabil 2022; 35(3): 655–674. 10.1111/jar.12977 35064736 PMC10119781

[32] United Nations Population Fund and Women Enabled International . The impact of COVID-19 on women with disabilities. UN, 2021.

[33] Bubela T Flood CM McGrail K , et al. How Canada’s decentralized COVID-19 response affected public health data and decision-making. BMJ 2023; 382: e075665. 10.1136/bmj-2023-075665 37487604

[34] Castro FP Kellison JG Boyd SJ , et al. A methodology for conducting integrative mixed methods research data analyses. J Mixed Methods Res 2010; 4(4): 342–360. 10.1177/1558689810382916 PMC3235529 22167325

[35] Lesko CR Fox MP Edwards JK . A framework for descriptive epidemiology. Am J Epidemiol 2022; 191(12): 2063–2070. 10.1093/aje/kwac115 35774001 PMC10144679

[36] Tong A Sainsbury P Craig J . Consolidated criteria for reporting qualitative research (COREQ): a 32-item checklist for interviews and focus groups. Int J Qual Health Care 2007; 19(6): 349–357. 10.1093/intqhc/mzm042 17872937

[37] Wang J Calderon G Hager ER , et al. Identifying and preventing fraudulent responses in online public health surveys: Lessons learned during the COVID-19 pandemic. PLOS Global Public Health 2023; 3(8): e0001452. 10.1371/journal.pgph.0001452 37610999 PMC10446196

[38] Arscott K Dagnan D Kroese BS . Consent to psychological research by people with an intellectual disability. J Appl Res Intellect Disabil 1998; 11(1): 77–83. 10.1111/j.1468-3148.1998.tb00035.x

[39] Patridge EF Bardyn TP . Research Electronic Data Capture (REDCap). J Med Library Assoc 2018; 106(1): 142–144. 10.5195/jmla.2018.319

[40] Harris PA Taylor R Thielke R , et al. Research electronic data capture (REDCap) – a metadata-driven methodology and workflow process for providing translational research informatics support. J Biomed Inform 2008; 42(2): 377–381. 10.1016/j.jbi.2008.08.010 18929686 PMC2700030

[41] Gundersen C Engelhard EE Crumbaugh AS , et al. Brief assessment of food insecurity accurately identifies high-risk US adults. Public Health Nutr 2017; 20(8): 1367–1371. 10.1017/S1368980017000180 28215190 PMC10261547

[42] Lowe B Wahl I Rose M , et al. A 4-item measure of depression and anxiety: Validation and standardization of the Patient Health Questionnaire-4 (PHQ-4) in the general population. J Affect Disord 2010; 122(1-2): 86–95. 10.1016/j.jad.2009.06.019 19616305

[43] Fitzgerald J Fitzgerald C . Using a simple mental health screen in vision rehabilitation needs assessment: The PHQ-4. Int J Ophthalmic Pract 2014; 5(4): 139–144. 10.12968/ijop.2014.5.4.139

[44] Gschwenter S Garber D Steininger B . Screening for anxiety and depression: Validation of the Patient Health Questionnaire for Depression and Anxiety (PHA-4) in a large sample of rehabilitation patients. Disabil Rehabil 2026: 1–15. 10.1080/09638288.2026.2649665 41915448

[45] Charmaz K . Constructing grounded theory. Sage Publications, 2006.

[46] Patton MQ . Enhancing the quality and credibility of qualitative analysis. Health Serv Res 1999; 34(5 Pt 2): 1189–1208. 10591279 PMC1089059

[47] Thomas DR . Feedback from research participants: Are member checks useful in qualitative research? Qual Res Psychol 2017; 14(1): 23–41. 10.1080/14780887.2016.1219435

[48] Althubaiti A . Sample size determination: A practical guide for health researchers. J Gen Fam Med 2022; 24(2): 72–78. 10.1002/jgf2.600 36909790 PMC10000262

[49] Braun V Clarke V . To saturate or not to saturate? Questioning data saturation as a useful concept for thematic analysis and sample size rationales. Qual Res Sport Exercise Health 2019; 13: 1–16. 10.1080/2159676x.2019.1704846

[50] Biggs MA Schroeder R Casebolt MT , et al. Access to reproductive health services among people with disabilities. JAMA Netw Open 2023; 6(11): e2344877. 10.1001/jamanetworkopen.2023.44877 38019515 PMC10687653

[51] Lamichhane A Rana S Shrestha K , et al. Violence and sexual and reproductive health service disruption among girls and young women during COVID-19 pandemic in Nepal: A cross-sectional study using interactive voice response survey. PLOS ONE 2021; 16(12): e0260435. 10.1371/journal.pone.0260435 34879111 PMC8654226

[52] Gul S Yagmur Y . The access of women with disabilities to reproductive health services during the COVID-19 pandemic: A Qualitative Study. Int J Caring Sci 2022; 15(2): 1242–1249.

[53] Evans M Rego A Ogbonna N , et al. Impacts of the COVID-19 pandemic on access to sexual and reproductive health services for women and transgender people with disabilities in Canada: A qualitative study. Sex Reprod Health Matters 2024; 32(1): 2441027. 10.1080/26410397.2024.2441027 39668741 PMC11730772

[54] Abacan A Raphael M . Exploring intersections and integration of sexual reproductive health and mental health among adolescents and young adults: Areas of collaboration and lessons learned. Bull Menninger Clinic 2023; 87(2): 105–114. 10.1521/bumc.2023.87.2.105 37260325

[55] Zatloff JP von Esenwein SA Philip Z , et al. Navigating a complex health system: The perceptions of psychiatric residents in addressing sexual and reproductive health of women with severe mental illness. Academic Psychiatr 2020; 44: 403–407. 10.1007/s40596-020-01197-x 32086796

