# Supplementary material for: Intersections of mental health and sexual and reproductive health during the COVID-19 pandemic: A mixed-methods study of women and gender-diverse people with disabilities
Source: Womens Health (Lond). 2026 Aug 10;22:17455057261466290. doi: 10.1177/17455057261466290 (PMC13458322; doi:10.1177/17455057261466290)
Supplement: Supplementary file 1 [file sj-pdf-1-whe-10.1177_17455057261466290.pdf]

Supplement A: Survey

Note: The survey data used in this study are part of a broader survey on the sexual and reproductive health experiences of people with disabilities during the COVID-19 pandemic. Only the modules relevant to the current study are included in this supplement.

Survey preamble

Thank you for agreeing to complete the "Disability and Reproductive Health during COVID-19" survey. We want to know about the impact of the COVID-19 pandemic on your reproductive health and health care. Reproductive health includes experiences related to contraception, abortion, sexually transmitted infections, cervical cancer screening, violence, pregnancy and postpartum health, and the health of your children (if applicable). We want to know whether the COVID-19 pandemic has impacted your reproductive health and access to services in these areas. We also want to know how these experiences have affected your mental health.

When we refer to the COVID-19 pandemic, we are talking about any time since March, 2020 until now.

The survey will take about 20 minutes to complete. If you do not want to answer a question, that is okay, just leave it blank. We thank you for your time!

Disability

The first few questions are about your disability and the supports you receive.

1. Do you have difficulty seeing, even if wearing glasses? Would you say:[1]
   - o No difficulty
   - o Some difficulty
   - o A lot of difficulty
   - o Cannot see at all

2. Do you have difficulty hearing, even if using a hearing aid(s)? Would you say:
   - o No difficulty
   - o Some difficulty
   - o A lot of difficulty
   - o Cannot hear at all

3. Do you have difficulty walking or climbing steps? Would you say:
   - o No difficulty
   - o Some difficulty
   - o A lot of difficulty
   - o Cannot do at all

[1] Questions 1-6 from: Washington Group on Disability Statistics. WG Short Set on Functioning. London: University College London; 2020.

4. Do you have difficulty remembering or concentrating? Would you say:
   - o No difficulty
   - o Some difficulty
   - o A lot of difficulty
   - o Cannot do at all

5. Do you have difficulty with self-care, such as washing all over or dressing? Would you say:
   - o No difficulty
   - o Some difficulty
   - o A lot of difficulty
   - o Cannot do at all

6. Using your usual language, do you have difficulty communicating, for example, understanding others or being understood? Would you say:
   - o No difficulty
   - o Some difficulty
   - o A lot of difficulty
   - o Cannot do at all

7. How would you describe your disability? What type of disability or disabilities do you have? [***free text***]

8. If you have a diagnosis for your disability, when did you first receive this diagnosis?
   - o Less than 2 years ago
   - o 2 years to less than 5 years ago
   - o 5 years to less than 10 years ago
   - o 10 or more years ago
   - o Not applicable; I have not received a diagnosis for my disability

9. Do you have any other physical or mental health conditions that impact you (e.g., diabetes, asthma, depression, anxiety)?
   - o No
   - o Yes (please specify): __________________

10. An assistive device is something made or changed to help a person do a task or activity. Do you use any kind of assistive device(s), for example, a wheelchair, cane, screen reader, tools, utensils or special grips?
   - o No
   - o Yes (please specify): __________________

11. **In the three months before the COVID-19 pandemic started**, did you get help with your daily activities (e.g., making meals, housework, bathing, taking medicine, scheduling, getting to appointments, doing errands) because of your disability? (check all that apply)
   - o No

- Yes, from a family member or friend
- Yes, from a service-provider (e.g., personal support worker)
- Other (please specify): _________________

12. ***If "yes" or "other" to #11:*** How often did you usually get this help?
    - Daily
    - At least once a week
    - At least once a month
    - Other (please specify): _________________

13. **Since the COVID-19 pandemic started,** have there been any changes to the help you get with your daily activities?
    - No
    - Yes, less help than before COVID-19
    - Yes, more help than before COVID-19

In the next set of questions, think about your relationships with friends, family members, coworkers, community members, and so on **during the COVID-19 pandemic**. Please indicate whether you agree each statement describes your support from other people.[2]

14. You have close relationships that provide you with a sense of emotional security and well-being.
    - Strongly disagree
    - Disagree
    - Agree
    - Strongly agree

15. There is someone you can talk to about important decisions in your life.
    - Strongly disagree
    - Disagree
    - Agree
    - Strongly agree

16. You have relationships where your strengths are recognized.
    - Strongly disagree
    - Disagree
    - Agree
    - Strongly agree

17. You feel part of a group of people who share your attitudes and beliefs.
    - Strongly disagree
    - Disagree
    - Agree
    - Strongly agree

[2] Questions 14-18 from: Orpana HM, Lang JJ, Yurkowski K. Validation of a brief version of the Social Provisions Scale using Canadian national survey data. Health Promot Chronic Dis Prev Can 2019;39(12):323-332.

18. There are people you can count on in an emergency.
- o Strongly disagree
- o Disagree
- o Agree
- o Strongly agree

[Note: Modules not relevant to the current study omitted, resulting in the omission of questions 19-123]

Mental health

The next set of questions is about your mental health.[3]

124. Over the past 2 weeks, how often have you been bothered by feeling nervous, anxious, or on edge?
- o Not at all
- o Several days
- o More than half the days
- o Nearly every day

125. Over the past 2 weeks, how often have you been bothered by not being able to stop or control worrying?
- o Not at all
- o Several days
- o More than half the days
- o Nearly every day

126. Over the past 2 weeks, how often have you been bothered by feeling little interest or pleasure in doing things?
- o Not at all
- o Several days
- o More than half the days
- o Nearly every day

127. Over the past 2 weeks, how often have you been bothered by feeling down, depressed, or hopeless?
- o Not at all
- o Several days
- o More than half the days
- o Nearly every day

128. **Before the COVID-19 pandemic,** how would you rate your overall mental health?

[3] Questions 124-127 from: Kroenke K et al. An ultra-brief screening scale for anxiety and depression: The PHQ-4. Psychosomatics 2009;50(6):613-621.

- Poor
- Fair
- Good
- Very good
- Excellent

129. How would you **currently** rate your overall mental health?
    - Poor
    - Fair
    - Good
    - Very good
    - Excellent

130. Have you sought care for your mental health since the COVID-19 pandemic started?
    - Yes
    - No

131. Has the COVID-19 pandemic made it hard for you to get or stopped you from getting mental health care? (check all that apply)
    - No → ***proceed to question #134***
    - Yes, I have had difficulty accessing a mental health specialist (e.g., psychiatrist)
    - Yes, I have had difficulty seeing my family doctor or nurse about my mental health
    - Yes, I have had difficulty accessing a community, student, or walk-in clinic about my mental health
    - Yes, I have had difficulty getting medication for my mental health
    - Yes, I have had difficulty accessing talk therapy or counselling services
    - Yes, I have had difficulty accessing a hospital or residential mental health treatment program
        a. Yes, other (please specify): __________________
        b. Not applicable; I have not sought mental health care since the COVID-19 pandemic started

132. ***If "Yes" to #131***: What has made it hard for you to get or stopped you from getting mental health care? (check all that apply)
    a. No transportation was available
    b. I was not able to leave the house
    c. I was afraid I might get COVID-19 and therefore did not want to go to the doctor / health centre
    d. Appointments with the doctor or health centre were not available
    e. Online or telephone appointments were not accessible
    f. Clinics or health centres were closed
    g. Clinics or health centres had long lines or waiting times
    h. I could no longer afford mental health care
    i. I did not have the regular supports or accommodations I needed to access this service
    j. Other (please specify): __________________

133. ***If "Yes" to #131***: Did this result in a delay in obtaining mental health care or prevent you from obtaining mental health care?
  a. No
  b. Yes, delayed a few days
  c. Yes, delayed 1-2 weeks
  d. Yes, delayed 3-4 weeks
  e. Yes, delayed more than 4 weeks
  f. Yes, I was prevented from obtaining mental health care

Socio-demographics

The remaining questions help us to understand your context.

134. How old are you? [***integer value***]

135. How do you describe your gender?
  - Cisgender woman
  - Transgender man
  - Transgender woman
  - Genderqueer or non-binary
  - Two-spirit
  - You don't have an option that applies to me. I identify as (please specify): _________________

136. What best describes your sexual identity or orientation?
  - Heterosexual/straight
  - Gay/lesbian
  - Bisexual
  - Queer
  - Two-spirit
  - You don't have an option that applies to me. I identify as (please specify): _________________

137. What best describes your relationship status?
  - Married or common-law
  - In a long-term relationship
  - Single, never married
  - Separated, widowed, or divorced
  - Other (please specify): _________________

138. In our society, people are often described by their race or racial background. For example, some people are considered "White" or "Black" or "East/Southeast Asian," etc. Which race category best describes you? (check all that apply)
  - Black (e.g., African, Afro-Caribbean, African-Canadian descent)
  - East/Southeast Asian (Chinese, Korean, Japanese, Taiwanese descent, Filipino, Vietnamese, Cambodian, Thai, Indonesian, other Southeast Asian descent)

- o Indigenous (e.g., First Nations, Inuit, Métis)
- o Latino (Latin American, Hispanic descent)
- o Middle Eastern (Arab, Persian, West Asian descent, e.g. Afghan, Egyptian, Iranian, Lebanese, Turkish, Kurdish, etc.)
- o South Asian (South Asian descent, e.g. East Indian, Pakistani, Bangladeshi, Sri Lankan, Indo- Caribbean, etc.)
- o White (European descent)

  You don't have an option that applies to me. I identify as (please specify): _________________

139. Were you born in Canada?
   - o Yes
   - o No. I was born in (please specify): _________________

140. ***If "no" to Question 139:*** How long have you lived in Canada?
   - o Less than 1 year
   - o 1 to 5 years
   - o More than 5 years

141. What is the highest level of education you have finished?
   - o Less than high school
   - o High school
   - o Trade school, college, CEGEP, or other non-university postsecondary training
   - o Undergraduate degree (bachelor's degree, e.g., BA, BSc)
   - o Graduate or professional degree (e.g., MSW, MPH, PhD, MD)

142. What part of Canada do you live in?
   - o Alberta
   - o British Columbia
   - o Manitoba
   - o New Brunswick
   - o Newfoundland and Labrador
   - o Northwest Territories
   - o Nova Scotia
   - o Nunavut
   - o Ontario
   - o Prince Edward Island
   - o Quebec
   - o Saskatchewan
   - o Yukon

143. Do you live in a …?
   - o Rural area (less than 1,000 people)
   - o Small town (between about 1,000 and 29,999 people)
   - o Medium-sized city (between about 30,000 and 99,999 people)
   - o Big city (between about 100,000 and 499,999 people)

- o Very big city (more than 500,000 people)

144. Which of the following **best** describes what you were doing **in the three months before the COVID-19 pandemic started**? (choose only one)
   - o Working full-time for pay
   - o Working part-time for pay
   - o Looking for paid work
   - o Volunteering
   - o Caring for family (e.g., children and/or an aging family member)
   - o On maternal/parental leave
   - o Going to school
   - o On medical or disability leave
   - o Other (please specify): _________________

145. ***If "working full-time for pay" or "working part-time for pay" in #144:*** **Since the COVID-19 pandemic started**, has your work status changed?
   - o No change: I continue doing the same work and going to the usual job site
   - o I keep doing the same work, but from home
   - o I keep doing the same work, but partly from home
   - o I am paid but unable to attend or do work
   - o I work on reduced time
   - o I lost my job
   - o I am temporarily unemployed
   - o I changed jobs
   - o Other (please specify): _________________

146. **Before the COVID-19 pandemic started, what was your annual household income before taxes?** (This amount includes money made by anyone living in your home and who helps with your and your family's costs. If you are currently on parental leave, think about how much money you made before your parental leave).
   - o $0 to $19,999
   - o $20,000 to $39,999
   - o $40,000 to $59,999
   - o $60,000 to $79,999
   - o $80,000 to $99,999
   - o $100,000 or more

147. **Since the COVID-19 pandemic started**, has your household experienced a loss of income?
   - o No loss of income
   - o Yes, a total loss of income
   - o Yes, a partial loss of income
   - o I had no personal income before COVID-19

148. **In the three months before the COVID-19 pandemic started,** what best described your housing situation?

- I lived alone
- I lived with roommates
- I lived with family (partner, children, other relatives)
- I lived in a group home
- I did not have stable housing
- Other (please specify): ________________

149. **Since the COVID-19 pandemic started**, have you experienced a change in your housing situation?
    - No
    - Yes (please specify): _________________

150. **Since the COVID-19 pandemic started**, have you worried that your food would run out before you got money to buy more?[4]
    - No
    - Yes, but less often than before COVID-19
    - Yes, but no more often than before COVID-19
    - Yes, more often than before COVID-19

151. **Since the COVID-19 pandemic started**, has the food you bought run out before you got money to buy more?
    - No
    - Yes, but less often than before COVID-19
    - Yes, but no more often than before COVID-19
    - Yes, more often than before COVID-19

Ending

152. Are there any other sexual, reproductive, maternal, and/or child health services that you have tried to access during the COVID-19 pandemic (e.g., related to transition, menstruation, or menopause)?
    _________________ [***free text***]

153. Is there anything else you would like to tell us about how the COVID-19 pandemic has affected your reproductive child health?
    _________________ [***free text***]

154. Did anyone help you complete the survey today?
    a. No
    b. Yes (please specify): _________________

155. Are you interested in participating in a follow-up interview on the same topic?
    a. No
    b. Yes

[4] Questions 150 and 151 from: Gundersen C et al. Brief assessment of food insecurity accurate identifies high-risk US adults. Public Health Nutr 2017;20(8):1367-71.

Thank you again for completing the survey. The Research Coordinator will be in touch with your $15 gift card. We wish you all the very best, and look forward to sharing the final study results with you in the future.
