# Supplementary material for: Intersections of mental health and sexual and reproductive health during the COVID-19 pandemic: A mixed-methods study of women and gender-diverse people with disabilities
Source: Womens Health (Lond). 2026 Aug 10;22:17455057261466290. doi: 10.1177/17455057261466290 (PMC13458322; doi:10.1177/17455057261466290)
Supplement: Supplementary file 2 [file sj-pdf-2-whe-10.1177_17455057261466290.pdf]

Supplement B: Interview Guide

Thank you for agreeing to be part of the "Disability and Reproductive Health during COVID-19" study. In this interview, we will be asking you questions about your experiences with sexual and reproductive health services during the COVID-19 pandemic (i.e., any service that you accessed during the pandemic or that you might have thought about getting, wanted to get or tried to get, accessed in different ways than usual, or had problems getting). When we refer to the "COVID-19 pandemic", we are talking about any time since March 2020 until now [i.e., date of interview ranging from May 2022 to March 2023].

Our questions include: (1) background questions about your disability and sexual and reproductive health history; (2) question about your experiences accessing sexual and reproductive health services during the COVID-19 pandemic; and (3) questions about how you think sexual and reproductive health services can be improved for people with disabilities. However, you do not have to answer questions. We want to hear your story and whatever experiences you'd like to share—we're interested in what you tell us is important. If you do not want to answer a question, you can just say "skip" or let us know in another way that works for you (is there a specific way you will let us know you want to skip a question?) We also know that topics related to reproductive health can be sensitive for some people. If you find a question is upsetting, please let us know how we can best support you. For example, we could skip the question, take a short break, or end the interview early—just let us know whatever you prefer.

Do you have any questions for us before we begin? Is it okay if we start audio recording?

1. How would you describe your disability or disabilities?

2. Do you have any other physical or mental health conditions that impact your life?

2. How do intersecting identities or experiences shape your experience with disability? *E.g., gender, sexuality, race, class, age.*

3. Can you tell us a little about your sexual and reproductive health history? *E.g., sexual education, sexual relationships, gender and sexuality, menstruation, sexually transmitted infections, cervical and breast cancer, contraception, abortion, pregnancy, fertility, childbirth, caring for young children, violence, mental health.*

4. Has your disability impacted your sexual and reproductive health over the years (or sexual life)?

5. Has the COVID-19 pandemic impacted your sexual and reproductive health (or sexual life)?

6. Can you tell us about any experiences when you needed sexual and reproductive health services during the COVID-19 pandemic? *E.g., related to menstruation, gynecology, sexually transmitted infection screening/care, cervical and breast cancer screening/care, contraception, abortion care, pregnancy-related care, fertility treatment, childbirth, health*

care for young children, menopause, gender affirmation, violence, mental health, or any other related services/therapies.

7. Did anything make it difficult or easier for you when seeking or accessing these services during the COVID-19 pandemic?

8. Did you get the support you needed for your sexual and reproductive health?

9. Do you think your experiences are similar to other people with disabilities?

10. Do you have any thoughts about how health care providers could make sexual and reproductive health services better for people with disabilities during a pandemic (or in general)?

11. Do you have any thoughts about how the government could make reproductive health services better for people with disabilities during a pandemic (or in general)?

12. Is there anything else about your experience that you want to share?
